# Supplementary material for: Bacteriophage SRD2021 Recognizing Capsular Polysaccharide Shows Therapeutic Potential in Serotype K47 Klebsiella pneumoniae Infections
Source: Antibiotics (Basel). 2021 Jul 22;10(8):894. doi: 10.3390/antibiotics10080894 (PMC8388747; doi:10.3390/antibiotics10080894)
Supplement: Supplementary file 1 [file antibiotics-10-00894-s001.zip › antibiotics-1251098-supplementary.pdf]

SUPPLEMENTAL MATERIALS

Table S1. Characteristics of 45 *Klebsiella pneumoniae* that cannot be lysed by ΦSRD2021

Strain	MLST[a]	Colony phenotype	Characteristics of bla[b]	Source[c]
A1876	ST1318	mucoid	NDM-1	Unknown
A1703	ST1333	mucoid	KPC-3	LRS
A1966	ST14	mucoid	IMP	Unknown
A1968	ST147	mucoid	NDM-1	Unknown
A2280	ST15	mucoid	KPC	LRS
A2282	ST15	mucoid	KPC	LRS
A2293	ST15	mucoid	KPC	Unknown
A1838	ST17	mucoid	IMP	Unknown
A1682	ST17	mucoid	negative	Blood
A2306	ST258	mucoid	KPC	Unknown
A2359	ST307	mucoid	IMP	LRS
A2368	ST307	mucoid	IMP	LRS
A1871	ST37	mucoid	negative	Urine
A2281	ST37	mucoid	NDM-1	LRS
A2612	ST395	mucoid	KPC	Unknown
A1851	ST395	mucoid	negative	Unknown
A1805	ST437	mucoid	NDM-1	Unknown
A1824	ST65	mucoid	KPC-2	Unknown
A2263	ST685	mucoid	KPC	LRS
A1749	ST709	mucoid	negative	Unknown
A1860	ST709	mucoid	IMP	Blood
A1683	ST846	mucoid	negative	Blood
A2366	ST86	mucoid	negative	Unknown
A2369	NEW	mucoid	IMP	AF
A2373	NEW	mucoid	KPC	LRS
A2371	NEW	mucoid	KPC	LRS
A1674	ST11	mucoid	KPC-2	Unknown
A1678	ND	mucoid	KPC-2	LRS
A1679	ST11	mucoid	KPC-2	LRS
A1705	ST449	mucoid	KPC-2, NDM-1	Urine
A1706	ST449	mucoid	KPC-2, NDM-1	LRS
A1763	ST11	mucoid	KPC-2	Unknown
A1831	ST11	mucoid	KPC-2	Unknown
A1836	ST11	mucoid	KPC-2	Unknown
A1850	ST40	mucoid	NDM-1	Unknown
A2314	ST11	mucoid	KPC-2	LRS
A2361	ND	mucoid	NDM-1	LRS
A2402	NEW	mucoid	KPC-2	Unknown
A2440	ND	mucoid	NDM-1	Blood
ATCC BAA 2146		mucoid	KPC	Urine
ATCC BAA 1899		mucoid	NDM	Unknown
ATCC BAA 1705		mucoid	ND	Urine
ATCC BAA 1706		mucoid	ND	Urine
A1680	ND	mucoid	NDM-1	Blood
A2307	ND	mucoid	NDM-1	Unknown

[a] MLST type: NEW, new ST types; ND, not determined; [b] Carbapenem antibiotic enzyme type: KPC, carbapenemases; NDM, New Delhi metallo-β-lactamase; IMP, imipenemase metallo-β-lactamase; [c] Clinical specimens source: AF, ascitic fluid; LRS, lower respiratory secretions.
